# Interrupted time series analysis for the impact of integrated medical insurance on direct hospitalization expense of catastrophic illness

**DOI:** 10.1038/s41598-022-15569-w

**Published:** 2022-07-19

**Authors:** Li Niu, Qiuhe Song, Yan Liu, Xin Wang

**Affiliations:** 1 Department of Preventive Medicine, Faculty of Medicine, Jiujiang University, Jiujiang, Jiangxi China; 2 Department of Dermatology, Affiliated Hospital, Jiujiang University, Jiujiang, Jiangxi China

**Keywords:** Health care economics, Health policy, Health services

## Abstract

In 2016, China initiated the merge of the urban resident basic medical insurance scheme and new rural cooperative medical scheme into one unified health insurance scheme: the urban and rural resident basic medical insurance. This study investigates the impact of integrated insurance on the direct hospitalization cost of inpatients with catastrophic illnesses. An interrupted time series analysis was conducted based on a sample of 6174 inpatients with catastrophic illness from January 2014 to December 2018. The factors surveyed included per capita total inpatient expense, out-of-pocket expense, and reimbursement ratio. Univariate analysis indicated that after the implementation of the unified urban and rural medical insurance, the reimbursed expense increased from 9398 to 13,842 Yuan (*P* < 0.001), average reimbursement ratio increased from 0.57 to 0.59 (*P* < 0.05). Expenses on both western and traditional medicines increased, although the proportion of medicine expense decreased after the integration. Interrupted time series analysis showed that per capita total inpatient expense and per capita out-of-pocket expense increased but showed a gradually decreasing trend after the integration. After the integration of urban and rural medical insurance, the average reimbursement ratio increased slightly, which had limited effect on the alleviation of patients’ financial burden. Furthermore, the integration effect on inpatient expense is offset by increased out-of-pocket medical expense due to suspected supplier-induced demand.

## Introduction

In China, the urban population increased to 56% in 2015, and more than 30% of the residents, or roughly 247 million, living in urban areas are migrants^1^. A new round of urbanization is underway in China on an unprecedented scale boosted by significant reform on household registration^2^. The long-lasting and fragmented urban–rural medical insurance systems has become a significant obstacle for migrant population to equally access health services and government subsidies^3,4^.

There were mainly three basic health insurance schemes in China: the new rural cooperative medical scheme (NCMS, which started in 2003), urban resident basic medical insurance scheme (URBMI, begun in 2007), and urban employee basic medical insurance scheme (UEBMI, launched in 1998). These schemes currently cover over 1.35 billion people (95% of the total population)^5^. People select health insurance schemes based on their employment status, household registration status (rural or urban), and living locations^6^. The benefit packages and financial protection are not identical within and across the schemes, hindering universal health insurance coverage in China. The annual per capita fund for UEBMI is approximately 6 and 7 times higher than that for the NCMS and URBMI, respectively^7^. Furthermore, NCMS is not transferable between provinces. The separated urban–rural health insurances have led to a greater financial burden for vulnerable populations such as rural to urban migrants.

To tackle the inequity caused by the separated insurance schemes, which vary in reimbursement and deductibles, NCMS and URBMI started to merge into one unified health insurance scheme—the urban and rural resident basic medical insurance (URRBMI) in 2016^8^. The current integration focuses on the unification of the capitation, deductible, reimbursement ratio, targeted population, drug catalogs, and the management systems^9^. The progress of the merge vary from province to province, and in many cities, the funds are still controlled at the county level^10^.

At present, the main goals of the integration are to increase actual coverage, reduce inequity, improve insurance transfer and unify management systems. However, little is known about the achievement of the goals^11,12^. In particular, few studies have investigated whether integrated medical insurance reduces out-of-pocket expenditure.

According to WHO, out-of-pocket expenditures in 2011 reached up to over 40% of total health expenditures in China^13^. For the past decade, the accelerated increase in public funding to subsidize health insurance in China has not lessened the out-of-pocket cost. The increased financial burden of healthcare has worsened the affordability of the vulnerable populations, especially those suffering from catastrophic illness in an underdeveloped region^14^. A catastrophic illness is a severe disease status requiring continued hospitalization or rehabilitation^15^. Catastrophic illness has been a substantial financial burden on patients and their families^16,17^.

This study investigates the impact of integrated urban and rural medical insurance on the direct hospitalization cost of inpatients with catastrophic illnesses. The findings would provide evidence for the improvements brought by URRBMI in China and offer experiences to other countries to tailor their fragmented medical insurance schemes.

## Methods

### Study region

The study was conducted at Jiujiang in Jiangxi province of China, which is located in southern China. The city has ten counties with a population of 4 million. Before 2016, patients covered by NCMS have reimbursed for all costs (including catastrophic medical insurance) before discharge^18^. Starting from 2016, integrated urban and rural medical insurance was implemented^19^. Furthermore, since October 2017, patients in the city were reimbursed for all benefits under a "one-stop instant reimbursement plan" before discharge, including benefits from basic medical insurance, catastrophic medical insurance, complement medical insurance for impoverished populations, and medical assistance from Civil Affairs Bureau^20^. In addition, local government extracts no less than 5% from the fund pool for catastrophic medical insurance which aims to provide extra reimbursements of high medical expenses^21^. The deductible of catastrophic medical insurance is determined as 50% of per capita disposable income of Jiujiang, the reimbursement ratio is 60%. The main differences and changes among NCMS and URRBMI in Jiujiang is listed in Table 1^22–25^.

**Table 1:** Comparison of NCMS and URRBMI between 2015 and 2018.

	NCMS 2015	URRBMI 2016	URRBMI 2017	URRBMI 2018
Premium (¥)	Individual: 90; Government: 320	Individual: 150; Government: 420	Individual: 180; Government: 450	Individual: 220; Government: 490
Capitation for basic insurance (¥)	100,000	30,000	100,000	100,000
Capitation for catastrophic insurance (¥)	250,000	250,000	250,000	250,000
Deductible (¥)	Primary hospital: 0; Secondary hospital: 400; Tertiary hospital: 600	Primary hospital:100; Secondary hospital: 200; Tertiary hospital: 300	Primary hospital:100; Secondary hospital: 400; Tertiary hospital: 600	All hospital: 400
Average reimbursement ratio for basic insurance (%)	Primary hospital: 90%; Secondary hospital: 80%; Tertiary hospital: 50%	Primary hospital: 75%; Secondary hospital: 65%; Tertiary hospital: 55%	Primary hospital: 90%; Secondary hospital: 80%; Tertiary hospital: 60%	All hospital: 80%
Reimbursement ratio for catastrophic insurance (%)	0–50,000: 50%; 50,000–100,000: 60%; > 100,000: 70%;	Primary hospital: 90%; Secondary hospital: 85%; Tertiary hospital: 80%	Primary hospital: 90%; Secondary hospital: 85%; Tertiary hospital: 80%	All hospital: 80%
Number of covered drugs	1100	2500	2500	2500

In this study, we chose one tertiary hospital out of six tertiary hospitals for analysis. The selected hospital was one of the largest hospitals with over 1500 beds.

### Data management

Data were retrieved from the hospital information system, containing information on all patients diagnosed with catastrophic illness admitted from January 2014 to December 2018. Catastrophic illness included the following 20 diseases and was covered by both URRBMI and Catastrophic Illness Medical Insurance: acute myocardial infarction, breast cancer, cerebral infarction, cervical cancer, chronic myelogenous leukemia, cleft lip and palate, colon cancer, colorectal cancer, end-stage kidney disease, esophageal cancer, gastric cancer, hemophilia, HIV opportunistic infection, hyperthyroidism, hypospadias, lung cancer, thalassemia, multidrug-resistant tuberculosis, severe mental illness, and type I diabetes^19^.

R package "tidyverse" was used for data cleaning. 5 years data were combined to a single file. 20 catastrophic illnesses were selected by matching the main diagnosis with ICD-10 code. The variables selected for the study included sociodemographic characteristics, length of stay, ICD-10 diagnosis, insurance, medical expense. For medical expense, outliers and missing data were explored. Complete cases were included to the final data. The consumer price index (CPI) of China from 2014 to 2018 was obtained from the World Bank^26^. All the medical costs were standardized by CPI taking the year 2014 as the reference.

### Data analysis

Interrupted time series analysis (ITSA) was used to analyze the effects of integration. ITSA is considered a robust quasi-experimental approach when control groups are lacking. In the interrupted time-series studies, segmented regression analysis is proven effective for estimating intervention effects^27–29^. In this study, the model was constructed as follows:

$${\text{Y}} \, = \, \beta_{0} + \, \beta_{{1}} \times {\text{ time }} + \, \beta_{{2}} \times {\text{ integration }} + \, \beta_{{3}} \times {\text{ time after integration }} + \, \varepsilon ,$$

where Y is the dependent variable representing the reduced percentage of per capita out-of-pocket inpatient expense, the per capita total inpatient expense, and the average reimbursement ratio. Time, integration, and time after integration was considered the independent variables. Time referred to each month from January 2014 to December 2018. Integration was assigned to 0 before the integration (January 2014 to December 2015) and one after the integration (January 2016 to December 2018). Time after integration was assigned to 0 before the integration and increased monthly after the integration (because there were 24 months before the integration and 36 after the integration, the value of this variable after the integration was set from 25 to 60). β_0_ is the level of explained variables at the beginning. β_1_ estimates the baseline trend before the integration. β_2_ estimates the effect of the integration on the change of intercept. β_3_ reflects the changed trend after the integration (the change in the slope). ε is the error term. All statistical analysis was conducted using R software version 3.5.2. R package "Wats" and "nlme" were used for ITSA, "ggplot2" was used for creating the graphs.

### Ethics declarations

The study was conducted according to the guidelines of the Declaration of Helsinki, and approved by the Ethics Committee of Jiujiang University (REC: JJU20160116).

Patient consent was waived by the Ethics Committee of Jiujiang University, because this study only involved de-identified secondary public access data.

## Results

**Figure 1:**
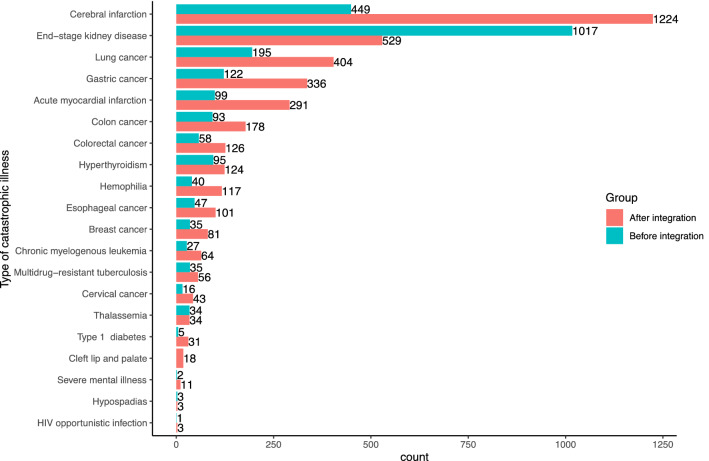
Comparison of catastrophic illnesses before and after the integration in the sampled hospital from 2014 to 2018.

The number of cases with any catastrophic illness before and after the integration in the sampled hospital from 2014 to 2018 is compared in Fig. 1. There were a total of 6174 cases, 2373 (38.6%) before the integration, 3774 (61.4%) after the integration. The top five were cerebral infarction, end-stage kidney disease, lung cancer, gastric cancer, and acute myocardial infarction.

After the integration of urban and rural medical insurance, the reimbursed expense increased from 9398 to 13,842 Yuan (*P* < 0.001), average reimbursement ratio increased from 0.57 to 0.59 (*P* < 0.05) (Table 2). Both per capita total inpatient expense and per capita out-of-pocket expense increased significantly, and comprehensive expense (general medical services, such as examination, bed and consultation, etc., and general treatment, such as nursing fees, injection, debridement and suture, dressing change, etc.), diagnostic expense, western medicine expense, and traditional medicine expense. The proportion of western medicine expense and traditional medicine expense decreased after the integration with significant difference.

**Table 2:** Main indicators for the integrated medical insurance in Jiujiang.

	Before integration (n = 2373) mean (SD)	After integration (n = 3774) mean (SD)	T-test (6145 df)	P value
Length of stay	24.42 (20.35)	17.78 (15.81)	14.302	< 0.001
Per capita total inpatient expense	16,599.41 (20,754.62)	22,874.16 (27,871.39)	9.443	< 0.001
Per capita out-of-pocket expense	6067.38 (8574.12)	8813.93 (9879.08)	11.156	< 0.001
Per capita reimbursed expense	9398.02 (13,224.05)	13,842.75 (20,986.35)	9.229	< 0.001
Average reimbursement ratio	0.57 (0.41)	0.59 (0.22)	2.129	0.0333
Comprehensive expense (CE)	3431.77 (6335.94)	4289.78 (9028.12)	4.045	< 0.001
CE proportion	0.26 (0.24)	0.19 (0.16)	14.011	< 0.001
Diagnostic expense (DE)	2810.37 (2789.24)	4424.76 (3259.52)	19.965	< 0.001
DE proportion	0.3 (2.07)	0.3 (0.34)	0.197	0.8441
Western medicine expense (WME)	6785.2 (9058.9)	7986.75 (10,845.4)	4.499	< 0.001
WME proportion	0.43 (0.74)	0.35 (0.2)	5.882	< 0.001
Traditional medicine expense (TME)	1206.05 (1869.53)	1620.31 (3106.12)	5.863	< 0.001
TME proportion	0.14 (1.23)	0.09 (0.13)	2.515	0.0119

**Figure 2:**
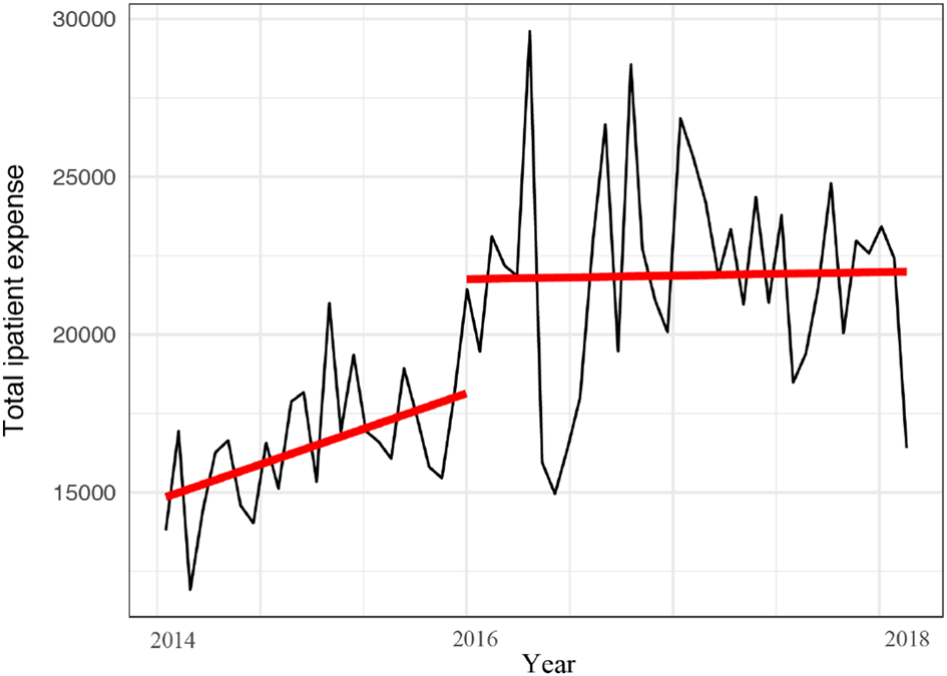
Trend of the per capita total inpatient expense (¥) from 2014 to 2018.

The ITSA indicated that the per capita total inpatient expense increased annually before the integration, but the increases were not statistically significant (Table 3 and Fig. 2). The per capita total inpatient expense increased by ¥3618.7 (*P* < 0.05) after the integration was implemented. After the integration, the slope of the per capita total inpatient expense decreased by ¥4.3 per month (*P* = 0.248).

**Table 3:** Interrupted time-series analysis of the impact of integrated medical insurance on the main indicators.

	β_1_	SE	P	β_2_	SE	P	β_3_	SE	P
Per capita total inpatient expense, ¥	4.510	3.200	0.164	3618.662	1747.832	0.043*	− 4.289	3.677	0.248
Per capita out-of-pocket expense, ¥	1.963	1.468	0.186	1265.567	787.672	0.114	− 1.224	1.717	0.479
Average reimbursement ratio, %	− 0.0002	0.00004	< 0.001***	0.066	0.021	0.002**	0.0002	0.00004	< 0.001***

*β* coefficient, *SE* standard error.

*, ** and *** denote P < 0.05, 0.01 and 0.001, respectively.

**Figure 3:**
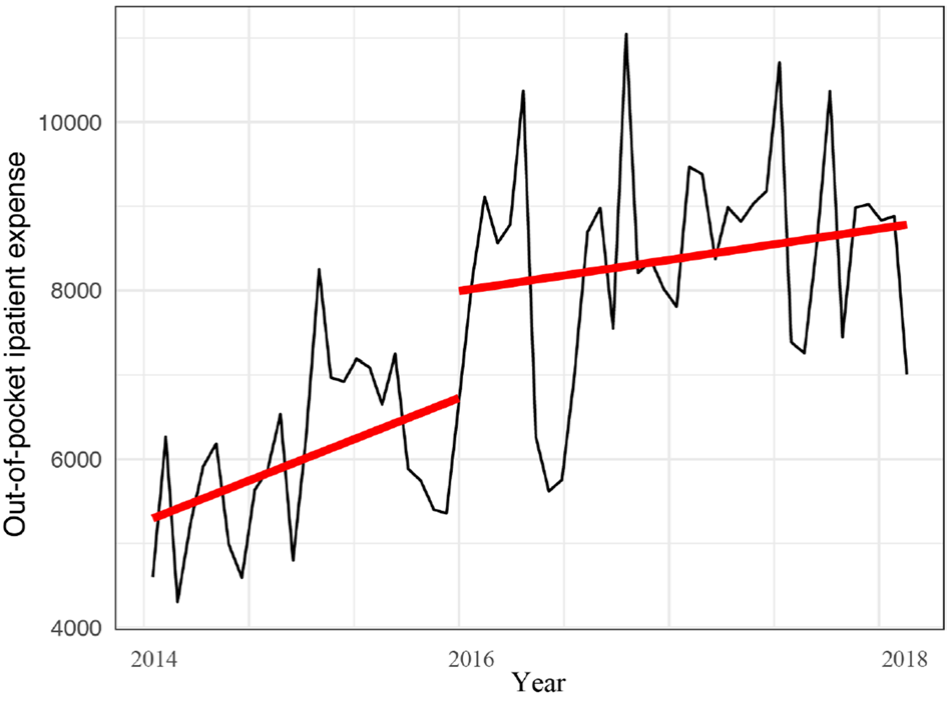
Trend of per capita out-of-pocket inpatient expense (¥) from 2014 to 2018.

The per capita out-of-pocket inpatient expense increased annually but insignificantly before the integration and increased by ¥1265.6 per month (P = 0.114) after the integration (Table 3 and Fig. 3). After the integration, the slope of the per capita out-of-pocket inpatient expense decreased by ¥1.2 per month (*P* = 0.479).

**Figure 4:**
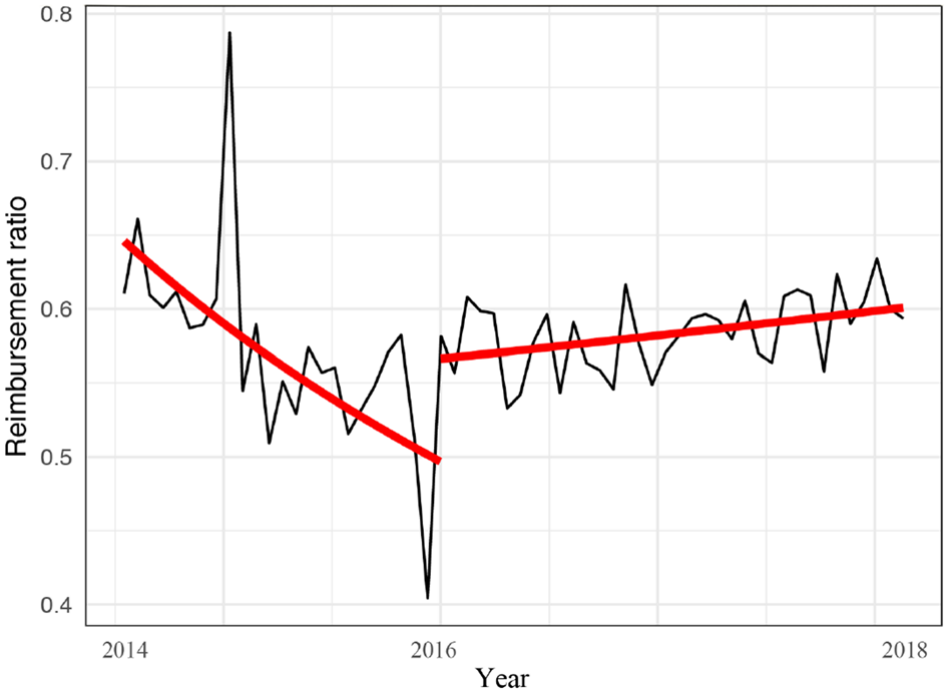
Trend of the average reimbursement ratio (%) from 2014 to 2018.

The average reimbursement ratio decreased monthly by 0.0002% per month before the reform (*P* < 0.001). As the reform was implemented, the average reimbursement ratio increased by 0.066% (*P* = 0.002) (Table 3 and Fig. 4). After the reform, the slope of the average reimbursement ratio increased by 0.0002% per month (*P* < 0.001).

## Discussion

This study evaluated the impact of URRBMI on the hospitalization expense of inpatients due to catastrophic illness in a city fully implemented with URRBMI. As our results showed that the steep decreasing trend of reimbursement ratio was halted and increased gradually right after the integration of urban and rural medical insurance, which was consistent with previous findings, individuals received more inpatient care benefits after the merge of urban and rural medical insurance^4^. However, our results indicated that the slightly increased average reimbursement ratio was much less than the designed 80% reimbursement ratio of URRBMI^23^, the actual average reimbursement ratio was still around 59% or even lower^30^. This is because not all medical services are covered by medical insurance, and the use of drugs outside the reimbursement catalog is not effectively controlled. Therefore, although the average reimbursement ratio is designed at a high level, it couldn't effectively reduce the medical burden of the insured unless it is achieved.

Our results showed that both per capita total inpatient expense and per capita out-of-pocket expense increase after the integration, but the increase is gradually reduced after the integration. To some extent, the integration of urban and rural medical insurance has triggered the release of the medical needs of rural residents^31^. For example, the average number of outpatient visits increased by 1.4 times, the total medical expenses increased by 35.3%, the inpatient expenses increased by 27.8%, and the outpatient medical expenses increased by 30.4% after the integration^30^. For patients with catastrophic illnesses, hospitalization and treatment with expensive drugs are needed, resulting in very high medical costs compared with the general population^32^.

In addition, this study revealed that both western and traditional medicine expenses increased, although the proportion of medicine expenses decreased after the integration. The integration of urban and rural medical insurance has not been able to standardize the profit of public hospitals. For instance, overtreatment due to supplier-induced demand, including unreasonable use of drugs and medical treatment still exists, resulting in increased per capita out-of-pocket expenses for inpatient visits^33^. Under the current "paying before treatment" plan, patients need to pay all or most of the medical expenses in advance, which further worsens their financial burden^34^.

## Conclusions

After the integration of urban and rural medical insurance, the average reimbursement ratio increased slightly, which had limited effect on the alleviation of patients’ financial burden. Furthermore, the integration effect on inpatient expense is offset by the increased out-of-pocket inpatient medical expense. Thus, policies should be adjusted to defer rising medical needs, use of non-covered drugs, and overtreatment.

## Limitations

Our study has some limitations. First, all cases were selected from one tertiary hospital in one city. The conclusions might be difficult to be generalized; Second, several critical independent variables, such as residents' and doctors' satisfaction with the integration and residents' health outcomes, were not included due to limited resources and time; Third, our findings could not entirely reflect the effectiveness of the integrated insurance, because we only showed the change of hospitalization expense, other essential aspects such as insurance coverage and patient satisfaction were not included. However, ITSA was applied in this study, which estimated the level of change after the integration and showed the changing trend that reflects the long-term effect of the integration. Our study provides novel evidence for this research area.

## Data Availability

The data that support the findings of this study are available from the corresponding author upon reasonable request.
